# Differences in Quality of Care Interactions Across Care Tasks in Assisted Living

**DOI:** 10.1093/geroni/igaf122.2040

**Published:** 2025-12-31

**Authors:** Rachel McPherson, Barbara Resnick, Sarah Holmes, Anju Paudel, Sorah Levy, Elizabeth Galik

**Affiliations:** University of Maryland School of Nursing, Baltimore, Maryland, United States; University of Maryland, Baltimore, Maryland, United States; University of Maryland School of Nursing, Baltimore, Maryland, United States; The Pennsylvania State University, University Park, Pennsylvania, United States; University of Maryland, Baltimore, Maryland, United States; University of Maryland School of Nursing, Baltimore, Maryland, United States

## Abstract

The quality of care interactions is crucial for optimizing the engagement of residents in personal care activities such as bathing and dressing. Limited research has explored how the quality of care interactions varies across different care tasks such as personal care (e.g., bathing, dressing), supportive/ambulatory care, or other care. As assisted living (AL) residents often require staff assistance in care tasks, it is critical to assess differences in the quality of care interactions that residents receive during the care tasks. This study aimed to determine whether the quality of care interactions differs by care task type in AL communities. This was a descriptive study done in four AL communities in Maryland. A total of 152 staff-resident care interactions were observed across various care tasks (e.g., intimate personal care, dietary care) using the Modified Quality of Interaction Scale. Descriptive statistics and a two-tailed analysis of variance with a post hoc Tukey HSD test were conducted. The majority of staff-resident interactions were positive (58%), and the remaining interactions were neutral (23%) or negative (19%). Most interactions occurred during supportive/ambulatory care (34%), followed by dietary care (24%), clinical care (21%), and intimate personal care (21%). There was a significant difference such that care interactions during supportive/ambulatory care were significantly more negative than interactions during personal care (p=.022). There were no other differences in quality of care interactions between care tasks. Findings from this study suggest that training AL staff on how to improve interactions during ambulation assistance or supportive care would be beneficial.

